# Interrater reliability of the modified Tinkertoy test: A validation study in schizophrenia and control groups

**DOI:** 10.1002/pcn5.70094

**Published:** 2025-04-03

**Authors:** Yasuhisa Nakamura, Reiko Miyamoto, Akihiro Koreki, Sachiko Anamizu, Masaru Mimura

**Affiliations:** ^1^ Department of Rehabilitation, Faculty of Health Sciences Nihon Fukushi University Aichi‐ken Japan; ^2^ Division of Occupational Therapy, Faculty of Health Sciences Tokyo Metropolitan University Tokyo Japan; ^3^ Department of Neuropsychiatry Keio University School of Medicine Tokyo Japan; ^4^ Department of Psychiatry NHO Tochigi Medical Center Tochigi Japan

**Keywords:** divergent thinking, executive function, interrater reliability, modified Tinkertoy test, schizophrenia

## Abstract

**Aim:**

Divergent thinking and executive function are critical components of cognitive performance, necessitating reliable assessment tools to guide clinical decision‐making and research on cognitive deficits. This study aimed to evaluate the interrater reliability of the modified Tinkertoy test (m‐TTT), a neuropsychological tool designed to assess these functions, particularly in individuals with schizophrenia.

**Methods:**

The interrater reliability of the m‐TTT was assessed in a sample of 40 Japanese participants, including 20 individuals with schizophrenia (12 males, eight females; mean age = 42.4 [standard deviation, 12.6] years) and 20 healthy controls (12 males, eight females; mean age = 40.0 [standard deviation, 9.6] years). Performances were independently scored by two occupational therapists using a standardized framework. Relative reliability was evaluated using intraclass correlation coefficients (ICCs), and absolute reliability was examined using Bland–Altman analysis.

**Results:**

In the schizophrenia group, ICC values indicated high interrater reliability for complexity (0.979), creation process (0.881), and total (0.969) scores. Similarly, in the control group, ICC values were high for complexity (0.969), creation process (0.790), and total (0.934) scores. Bland–Altman analysis demonstrated no fixed or proportional bias; however, greater variability was observed at higher creation process scores in the control group.

**Conclusion:**

The results confirm the high interrater reliability of the m‐TTT, supporting its utility as a robust tool for assessing cognitive deficits and guiding rehabilitation strategies in psychiatric contexts. However, the study's generalizability is limited by its Japanese‐only sample, necessitating further validation across diverse populations and cultural settings.

## Graphical abstract

This study evaluated the interrater reliability of the modified Tinkertoy test (m‐TTT) in schizophrenia and control groups. Results demonstrated high reliability and highlighted the tool's potential for assessing cognitive deficits in clinical settings.

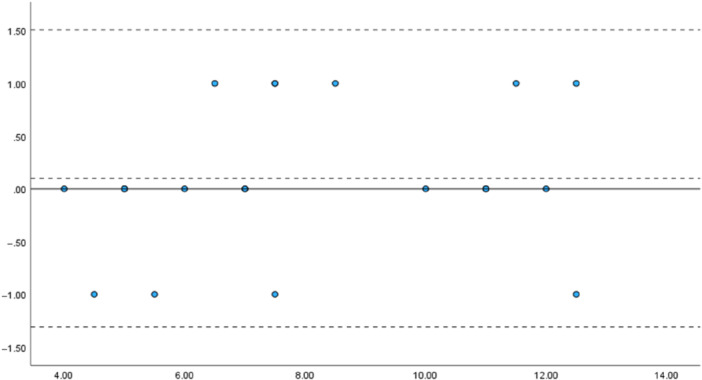

## INTRODUCTION

Executive function and divergent thinking are critical cognitive domains that are extensively studied in neuropsychology to understand the cognitive processes underlying creativity, problem‐solving, and adaptive planning.^1^, ^2^, ^3^ These domains hold particular importance in clinical evaluations, as impairments can profoundly impact daily functioning and diminish the quality of life in individuals with neurological or psychiatric disorders.^4^, ^5^, ^6^ Moreover, contemporary research highlights the interconnectedness of executive function and creativity, particularly in clinical populations such as those with schizophrenia, where cognitive deficits complicate adaptive problem‐solving and real‐world functioning.^7^

Various assessment tools have been developed to evaluate these constructs, with task‐based measures gaining prominence because of their ability to simulate real‐world applications. Among them, the Tinkertoy test (TTT) has emerged as a practical and ecologically valid tool for assessing divergent thinking and executive function, making it widely used in research and clinical practice.^8^, ^9^ However, the ability to capture the multifaceted cognitive processes involved in creative thinking and task execution in the traditional TTT scoring system is limited.^10^, ^11^, ^12^ Moreover, standard scoring approaches often cannot adequately reflect abilities, such as generating novel ideas, adapting strategies, or integrating feedback, which are crucial for assessing real‐world functional outcomes. These shortcomings are particularly problematic in populations with conditions such as schizophrenia, in which traditional measures often lack predictive validity for daily living skills.^7^

The modified Tinkertoy test (m‐TTT) was developed to address these limitations. This test incorporates additional scoring criteria, including the creation process score, to enhance the test's sensitivity in evaluating divergent thinking. The scoring criteria for the m‐TTT have been evaluated in terms of internal consistency and criterion‐related validity in relation to divergent thinking tests.^13^ This updated framework provides a more comprehensive analysis of cognitive processes and offers valuable insights into individual strengths and deficits. Preliminary studies have indicated that the m‐TTT correlates more strongly with daily living skills in individuals with schizophrenia than do conventional assessments of divergent thinking, including the original TTT.^14^ However, despite its potential, the interrater reliability of the m‐TTT scoring system remains insufficiently explored. Comparisons with the reliability standards established for other neuropsychological assessments further underscore this gap.^15^

Reliability is a fundamental aspect of psychometric evaluations that ensures consistent and reproducible outcomes across evaluators and contexts. Specifically, interrater reliability plays a vital role in validating scoring procedures by demonstrating consistent application of the criteria among independent evaluators. The absence of strong interrater reliability undermines the effectiveness of the m‐TTT as a diagnostic and predictive tool. Notably, the original TTT has not undergone formal validation of interrater reliability, highlighting a critical gap in its psychometric evaluation. Addressing this issue is particularly significant in schizophrenia, where reliable assessments are crucial for designing personalized rehabilitation strategies.^16^ Recent methodological advances emphasize the importance of establishing robust reliability metrics to ensure the ecological validity of assessments in clinical and research settings.^15^

By establishing a standardized scoring framework, this study aimed to enhance the reliability and utility of the m‐TTT as a tool to assess divergent thinking and executive function. To achieve this, the interrater reliability of the m‐TTT was evaluated by analyzing video recordings of participants with schizophrenia, as well as healthy controls, completing the test. Two independent raters scored these videos using the m‐TTT criteria to rigorously assess both relative reliability (consistency in rankings across raters) and absolute reliability (precision of individual scores).

## METHODS

### Participants and recruitment

Participants with schizophrenia were recruited from a psychiatric day care center via poster advertisements between January and December 2022, while healthy controls were identified using the snowball sampling method during the same period. All participants were provided with both written and verbal explanations of the study's purpose, and informed consent was obtained before data collection.

This study included two groups: the schizophrenia group, consisting of individuals with schizophrenia; and the control group, consisting of healthy individuals. All participants in the schizophrenia group had a confirmed diagnosis of the disorder based on the *Diagnostic and Statistical Manual of Mental Disorders*, Fifth Edition, Text Revision (DSM‐5‐TR)^17^ criteria and were receiving outpatient care, including pharmacological treatment and attendance at psychiatric daycare facilities. Participants with schizophrenia were included in the study if they had stable living conditions and no severe physical or intellectual disabilities.

Healthy controls were recruited through personal networks and were screened to confirm no personal or family history of psychiatric disorders. All participants in both groups were right‐handed, as determined using the Edinburgh Handedness Inventory.^18^

A video of the m‐TTT administration process was recorded after obtaining consent from all participants.

### Exclusion criteria

Exclusion criteria for all participants included a history of intellectual disability, significant neurological injuries (e.g., traumatic brain injury or stroke), or a diagnosis of substance use disorder within the past 6 months based on the DSM‐5‐TR criteria.

### Participant flow

The initial pool consisted of 52 potential participants, including 31 individuals with schizophrenia and 21 healthy controls. Following eligibility screening, 12 participants were excluded either for not meeting the selection criteria or for withdrawing consent. Specifically, eight individuals with schizophrenia did not meet the eligibility criteria, and three withdrew consent, while one healthy control participant also withdrew consent. The final sample included 20 participants in each group.

To ensure an adequate sample size, we calculated the required sample size based on Donner and Eliasziw's method^19^ for intraclass correlation coefficient (ICC) analysis, which was employed to assess interrater reliability. With an expected ICC of 0.8 and a margin of error set at ±0.111, the analysis indicated that a minimum sample size of 19 participants would be sufficient. In this study, we included 20 participants with schizophrenia and 20 healthy controls, totaling 40 participants. This sample size exceeds the required threshold, ensuring robust statistical power for the analysis.

### Evaluators

The evaluators, two male occupational therapists in their 30s, had 11 years (Evaluator A) and 15 years (Evaluator B) of clinical experience. To ensure consistent application of the scoring criteria, both evaluators reviewed the scoring manual for the m‐TTT and completed standardized training, which included guided practice sessions with example cases and feedback from a researcher with expertise in the m‐TTT scoring criteria. This preparatory process was conducted to enhance the reliability of the evaluations by minimizing subjective variability and ensuring adherence to a standardized framework.

### Modified Tinkertoy test

This study utilized the m‐TTT, a modified version of the original TTT. A Tinkertoy is a construction toy set made up of wooden pieces designed for children. Originally, the TTT was developed as a measure of executive functioning. However, the m‐TTT expands on this by introducing a novel creation process score that emphasizes the free‐composition aspect of the task and evaluates its utility as a test of divergent thinking.

For the m‐TTT, the participants were instructed to construct a structure using 50 freely assembled pieces from the Tinkertoy set. The sole directive provided was to “Make whatever you want.” The participants were required to spend at least 5 min completing the task and to independently declare when their work was completed. If the participants finished the task in less than 5 min, they were encouraged to continue reflecting on their creations.

The unstructured nature of the test allows participants to initiate, plan, and execute complex tasks autonomously. The criterion‐related validity of the cumulative m‐TTT score is supported by its correlation with both the verbal and nonverbal divergent thinking scores.

The creation process score demonstrates internal consistency with traditional evaluation criteria, emphasizing that the m‐TTT is not merely an amalgamation of separate scoring systems (complexity and creation process scores).^13^ Instead, these components represent the interconnected dimensions of divergent thinking, yielding enhanced accuracy in assessing divergent thinking. Upon completion of the task, participants were asked to describe their construction. If a structure corresponded to a specific object, it was evaluated based on the appropriateness of its alignment with the provided name or concept. The follow‐up questions included: “How did you approach the task after observing the pieces?,” “How did you adapt if your original plan did not work?,” and “What does each part of your structure represent?” The examiner used the participants' responses, along with observational data, to evaluate the creation process.

The original scoring system assigns points based on seven criteria, as summarized in Table 1. The complexity score (Comp) is the sum of these variables. The creation process score is derived as follows: a score of 0 indicates no clear creative vision or understanding of the construction, whereas a score of 1 indicates limited creative intent, resulting in partial completion. A score of 2 denotes the development of a creative idea during the task, culminating in a complete structure. A score of 3 indicates a clear creative vision from the outset, followed by completion. A score of 4 represents not only a clear initial vision but also the consideration of the environmental context within the construction. The total m‐TTT score is calculated by combining the complexity and creation process scores.

**Table 1:** Modified Tinkertoy test scoring criteria.

Variable	Scoring criteria	Points
1. MC	Made constructions: Created using any combination of pieces ＝ 1	1
2. NP	Number of pieces used (*n*) 0–19 ＝ 1, 20–39 ＝ 2, 40–49 ＝ 3, Exactly 50 ＝ 4 Note: “*n*” denotes the total number of parts used in the creation process	1–4
3. Name	Appropriate ＝ 3, vague/inappropriate ＝ 2, post‐hoc naming, description ＝ 1, none ＝ 0	0–3
4. MOV	Mobility ＝ 1, moving parts ＝ 1	0–2
5. 3D	Three‐dimensional	1
6. Stand	Free‐standing, stays standing	1
7. Errors	For each error (misfit, incomplete fit, dropped and not picked up)	–1
8. Scoring for creation process	The participant did not have a creation image envisaged and completed or did not know what was created with Tinkertoy ＝ 0. The participant was unable to create with a clear image and completed a portion of some kind of work ＝ 1. The participant created images in the process of assembly, created work, and finished it ＝ 2. The participant created images, created work, and finished it ＝ 3. The participant created and completed the work based on the surrounding environment ＝ 4.	0–4
Highest score possible		16
Lowest score possible		−1 or less

*Note*: MC, made constructions; NP, number of pieces used; Name, naming appropriateness of the structure; Mob, functional mobility, such as operational wheels; MOV, presence of moving parts; 3D, three‐dimensional complexity; Stand, freestanding stability; Errors, performance errors, including misfitting parts, incomplete connections, or dropped pieces left unrecovered.

### Scoring conditions and environment

**Figure 1:**
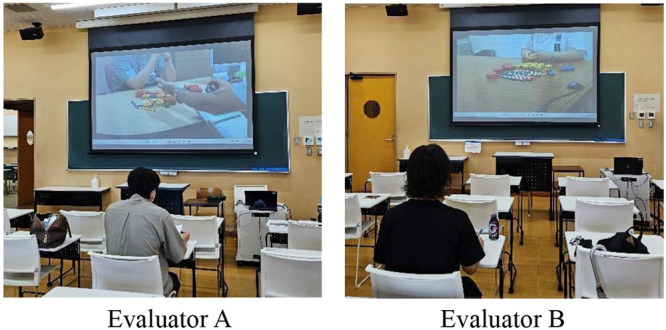
Evaluator scoring conditions.

The m‐TTT videos were scored in a lecture room at the university (Figure 1). Two evaluators independently assessed the participants' performance separately by reviewing video recordings projected onto a screen. Printed transcripts of the post‐test interviews were provided to supplement the evaluation. The number of components used during the test was specified to guide the scoring. The video duration ranged from 360 to 1200 s per test scenario.

### Statistical analysis

The relative and absolute reliabilities of the m‐TTT measurements determined by the two evaluators were analyzed for both the schizophrenia and control groups. Relative reliability was evaluated using the ICC with 95% confidence intervals (CIs), whereas absolute reliability was assessed using the Bland–Altman analysis to identify the presence, type, and range of measurement errors.

In the Bland–Altman analysis, the *y*‐axis represented the difference between the two evaluators' measurements, and the *x*‐axis represented the mean of the measurements, generating a Bland–Altman plot to detect systematic errors. Additive bias was assessed using a one‐sample *t*‐test for mean differences in the measurements. Additive bias was defined as a *p* < 0.05 and a 95% CI excluding 0.

Proportional bias was evaluated using simple linear regression, with measurement differences and mean as the dependent and independent variables, respectively. A statistically significant regression slope (*p* < 0.05) indicated a proportional bias. A scatterplot was created to visualize the differences in scores relative to the score range.

To compare m‐TTT scores between the schizophrenia and healthy control groups, an independent *t*‐test (or Mann–Whitney *U*‐test, if normality was not met) was conducted for the scores of each evaluator.

Additionally, we performed a correlation analysis to examine the relationships between m‐TTT scores, educational attainment, antipsychotic medication dosage, and psychiatric symptoms. Pearson's correlation coefficients (or Spearman's rank correlation coefficients if normality was not met) were calculated between m‐TTT scores, Positive and Negative Syndrome Scale (PANSS) scores (Positive Symptoms, Negative Symptoms, and General Psychopathology), chlorpromazine‐equivalent antipsychotic dosage, and educational attainment.

All statistical analyses were conducted using IBM SPSS Statistics for Windows (Version 27.0; IBM Corp.).

## RESULTS

### Comparison of basic information between the schizophrenia and control groups

This study included 40 participants comprising 20 individuals with schizophrenia (12 males, eight females) and 20 healthy controls (12 males, eight females). The schizophrenia group had a mean age of 42.4 ± 12.6 years, a mean educational attainment of 13.9 ± 1.8 years, a Japanese Adult Reading Test (JART)‐estimated intelligence quotient (IQ) of 98.9 ± 10.0, and a mean chlorpromazine‐equivalent antipsychotic dose of 616.2 ± 241.0 mg. The PANSS scores for the schizophrenia group were as follows: Positive Symptoms, 19.7 ± 3.4; Negative Symptoms, 19.0 ± 5.0; and General Psychopathology, 38.7 ± 5.5.

The control group had a mean age of 40.4 ± 9.6 years, a mean educational attainment of 14.9 ± 1.3 years, and a JART‐estimated IQ of 102.2 ± 6.7. No statistically significant differences between the groups were observed in terms of age, sex, number of years of education, or estimated IQ as measured using the JART‐estimated IQ (all *p* > 0.05).

### Scoring results of the evaluators

Table 2 summarizes the m‐TTT complexity, creation process, and total scores recorded by Evaluators A and B for the schizophrenia and control groups.

**Table 2:** Modified Tinkertoy test scoring results by evaluator for each group.

	Schizophrenia group		Healthy control group	
	Evaluator A	Evaluator B	Evaluator A	Evaluator B
Complex	6.6 ± 1.9	6.7 ± 1.9	8.3 ± 1.6	8.2 ± 1.5
Creation process	1.5 ± 1.3	1.5 ± 1.1	2.6 ± 0.9	2.3 ± 0.9
Total	8.2 ± 2.9	8.1 ± 2.8	10.8 ± 2.2	10.5 ± 2.1

*Note*: Values are presented as mean ± standard deviation.

### Interrater reliability

#### Relative reliability: ICC

For the schizophrenia group, Evaluators A and B demonstrated ICC values of 0.979 (95% CI: 0.950–0.991), 0.881 (95% CI: 0.734–0.950), and 0.969 (95% CI: 0.927–0.987) for the complexity, creation process, and total scores, respectively. For the control group, the ICC values were 0.969 (95% CI: 0.924–0.987), 0.808 (95% CI: 0.591–0.917), and 0.940 (95% CI: 0.861–0.975) for the complexity, creation process, and total scores, respectively.

#### Absolute reliability: Bland–Altman analysis and the assessment of fixed and proportional bias

**Figure 2:**
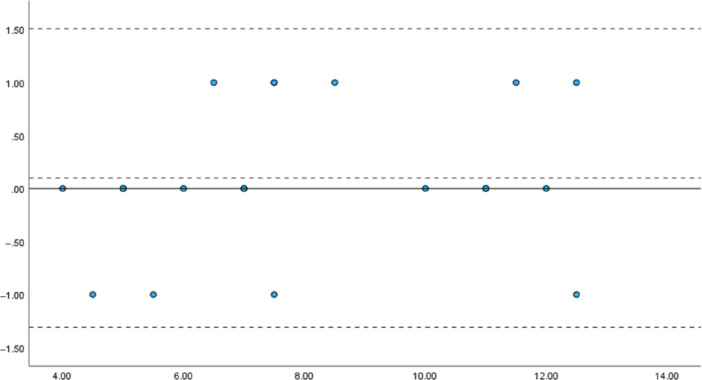
Bland–Altman analysis of modified Tinkertoy test (m‐TTT) total scores in the schizophrenia group. *Note*: *x*‐axis: Mean m‐TTT total scores, *y*‐axis: Differences in the m‐TTT total scores.

**Figure 3:**
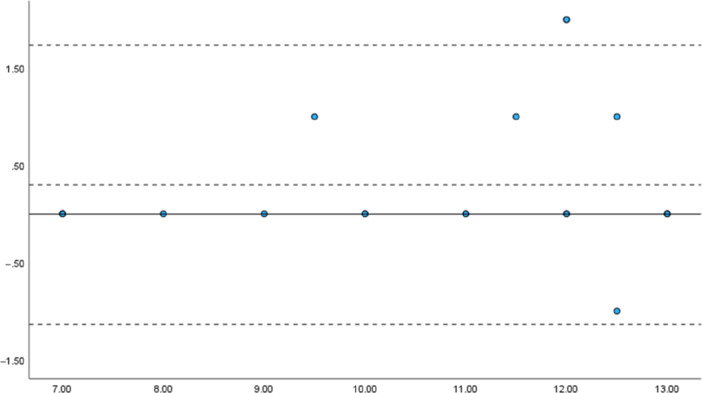
Bland–Altman analysis of modified Tinkertoy test (m‐TTT) total scores in the healthy control group. *Note*: *x*‐axis: Mean m‐TTT total scores, *y*‐axis: Differences in the m‐TTT total scores.

The results of the Bland–Altman analysis of the m‐TTT total scores, based on evaluations by Evaluators A and B, are shown in Figure 2 for the schizophrenia group and in Figure 3 for the control group. For the schizophrenia group, the *t*‐test revealed no fixed bias (*p* = 0.54; 95% CI: −0.41 to 0.21). A simple regression analysis indicated a slope of 0.014 (*p* = 0.81), confirming the absence of proportional bias. For the control group, a one‐sample *t*‐test of the total score also showed no fixed bias (*p* = 0.14; 95% CI: −0.853 to 0.585). A simple regression analysis revealed a slope of 0.049 (*p* = 0.55), confirming the absence of proportional bias.

#### Comparison of demographic data and m‐TTT scores between the schizophrenia group and the healthy control group

Table 3 presents a comparison of demographic data and m‐TTT scores between the schizophrenia and healthy control groups. No significant differences were observed in demographic data between the groups. The schizophrenia group showed significantly lower m‐TTT scores in all components: complex, creation process, and total scores.

**Table 3:** Demographic and modified Tinkertoy test score comparison between schizophrenia and control groups.

Measure	Group	Median(IQR)/Mean ± SD	*p* value	Effect size (*r* /Cohen's *d*)
Age	Schizophrenia	42.0 ± 12.8	0.65	0.15
	Healthy control	40.4 ± 9.6		
Education	Schizophrenia	13.5 (12–16)	0.08	−0.32
	Healthy control	15.0 (14–16)		
JART IQ	Schizophrenia	99 (94.1–109.3)	0.41	−0.16
	Healthy control	102 (99.3–107)		
Complex score; Evaluator A	Schizophrenia	6.5 (5.0–8.0)	0.006	−0.51
	Healthy control	8.5 (7.0–9.0)		
Complex score; Evaluator B	Schizophrenia	6.5 (5.0–8.3)	0.009	−0.88
	Healthy control	8.5 (7.0–9.0)		
Creation process score; Evaluator A	Schizophrenia	1.5 (0–2.4)	0.008	−0.48
	Healthy control	3.0 (2.0–3.0)		
Creation process score; Evaluator B	Schizophrenia	1.0 (1.0–2.0)	0.009	−0.47
	Healthy control	2.0 (2.0–3.0)		
Total score; Evaluator A	Schizophrenia	7.5 (5.8–11.0)	0.004	−0.53
	Healthy control	11.5 (9.8–13.0)		
Total score; Evaluator B	Schizophrenia	7.0 (6.0–11.0)	0.005	−0.52
	Healthy control	11.0 (9.0–12.0)		

Figure S1 presents examples of structures created by participants in both groups, illustrating differences in complexity and design approach. The schizophrenia group tended to create simpler and less varied structures compared to the healthy control group.

#### Correlation analysis of m‐TTT, educational attainment, chlorpromazine‐equivalent antipsychotic dose, and PANSS

Table 4 presents the correlation analysis of m‐TTT scores with educational attainment, chlorpromazine‐equivalent antipsychotic dose, and PANSS subscales. Significant negative correlations were observed between PANSS Positive Symptoms and Evaluator A creation process scores (*ρ* = −0.47, *p* < 0.05), Evaluator B creation process scores (*ρ* = −0.46, *p* < 0.05), and Evaluator A total scores (*ρ* = −0.44, *p* < 0.05).

**Table 4:** Correlation analysis between modified Tinkertoy test scores, antipsychotic dose equivalents, and PANSS scores.

Measure	Educational attainment	Chlorpromazine‐equivalent antipsychotic dose	PANSS		
			Positive Symptoms	Negative Symptoms	General psychopathology
Complex score; Evaluator A	0.09	0.39	−0.39	0.01	0.34
Complex score; Evaluator B	0.09	0.41	−0.40	−0.01	0.24
Creation process score; Evaluator A	0.31	0.36	−0.47*	−0.01	0.04
Creation process score; Evaluator B	0.24	0.36	−0.46*	−0.01	−0.06
Total score; Evaluator A	0.12	0.40	−0.44*	0.01	0.19
Total score; Evaluator B	0.21	0.39	−0.41	0.07	0.19

Abbreviation: PANSS, Positive and Negative Syndrome Scale.

* *p* < 0.05.

## DISCUSSION

This study evaluated the interrater reliability of the m‐TTT, a neuropsychological instrument for assessing divergent thinking and executive functioning. The findings demonstrated the high reliability of the m‐TTT scoring system, highlighting its potential to address the limitations of traditional assessment methods in clinical and research settings.

Interrater reliability, a key psychometric property ensuring consistency across evaluators, was strong in both groups. The schizophrenia group showed high clarity and reproducibility in scoring criteria, even among individuals with cognitive impairments. The control group also exhibited strong reliability; however, slightly lower consistency in evaluating the creation process suggested the inherent subjectivity of scoring creative tasks. Despite this variability, interrater reliability remained within clinically acceptable ranges. Bland–Altman analysis further confirmed the reliability, with no fixed or proportional bias in either group. These findings align with prior research, reinforcing the importance of robust interrater reliability for the usability and validity of neuropsychological tools.^19^, ^20^

In the control group, higher m‐TTT scores were associated with increased score dispersion, particularly in the creation process evaluation. Subjective judgment contributed to variability, suggesting that elevated creativity levels may challenge interrater consistency. This finding is consistent with prior studies that highlight the difficulties in scoring creativity due to subjective interpretation.^21^, ^22^ Although the m‐TTT demonstrated strong reliability, refining the scoring criteria or enhancing evaluator training may improve consistency, particularly in highly creative responses.

Comparisons between the schizophrenia and control groups revealed significant differences in m‐TTT performance. No significant differences were found in age, education, or estimated IQ, indicating that cognitive performance differences were not attributable to these demographic factors. However, the schizophrenia group exhibited significantly lower scores across all components—complex, creation process, and total scores—suggesting impairments in visuospatial constructional abilities and creative processing. These deficits align with previous research linking schizophrenia to executive dysfunction and impaired divergent thinking.^14^ The m‐TTT requires participants to generate, plan, and assemble structures using predefined materials, engaging higher‐order cognitive processes, such as working memory, problem‐solving, and motor coordination. The observed deficits may reflect prefrontal cortex dysfunction, a region heavily involved in executive function and divergent thinking.

Correlation analysis revealed a modest negative association between PANSS Positive Symptoms and m‐TTT performance, particularly in the creation process and total scores. While greater severity of positive symptoms, such as hallucinations and delusions, showed some relationship with reduced engagement in structured creative processes, the strength of this association was not pronounced. Prior studies have reported links between positive symptoms and difficulties in set‐shifting, reasoning, and problem‐solving,^23^, ^24^ which may partly explain the observed trend. Given the freely constructed nature of the m‐TTT, which engages cognitive flexibility, reasoning, and problem‐solving, it is possible that schizophrenia symptomatology influences performance to some extent. However, further research is needed to clarify the extent and nature of this relationship.

Traditional divergent thinking assessments, such as verbal fluency and paper‐and‐pencil tasks, have been criticized for limited ecological validity.^25^, ^26^ The m‐TTT addresses these limitations by incorporating additional scoring criteria, including the creation process score, which evaluates cognitive processes during task performance rather than focusing solely on the final product. This feature is particularly valuable for individuals with schizophrenia, who often exhibit deficits in planning, problem‐solving, and adaptive creativity that impair daily functioning.^13^, ^27^ The creation process score captures essential cognitive elements, such as strategy adaptation, integration of novel components, and problem‐solving flexibility, enabling a more comprehensive assessment of cognitive function.

The findings support the m‐TTT as a reliable tool for clinical and research applications. In clinical settings, the m‐TTT may aid in assessing cognitive deficits and guiding rehabilitation strategies. By capturing detailed aspects of executive functioning and divergent thinking, it provides valuable insights for personalized therapeutic interventions. For example, m‐TTT data could inform targeted rehabilitation programs aimed at improving problem‐solving and planning abilities, ultimately enhancing daily life functioning. In research settings, the standardized scoring system and demonstrated reliability ensure consistency across studies, facilitating the accumulation of cohesive evidence on divergent thinking and executive functioning.

Several strengths enhance the significance of this study. The rigorous validation of the m‐TTT scoring criteria confirmed high interrater reliability in both groups, demonstrating clarity and reproducibility even among individuals with cognitive impairments. The inclusion of participants with stable psychiatric conditions, recruited from outpatient treatment programs and psychiatric day care facilities, ensured that findings are applicable to real‐world clinical contexts. Additionally, the structured and reproducible scoring system addressed the challenge of assessing creativity, a domain often criticized for subjectivity.

Despite these strengths, certain limitations should be considered. The relatively small sample size, determined with a margin of error set at ±0.111, ensured feasibility but slightly reduced the precision in ICC estimation. Participants were recruited from psychiatric day care facilities and outpatient programs, representing populations with stable living conditions and regular psychiatric care.

To confirm the adaptability of the m‐TTT, replication in different settings—such as hospitals, home‐based care, and rehabilitation centers—is necessary. Expanding the sample to include a broader range of cultural, ethnic, and socioeconomic backgrounds would enhance generalizability and validate the m‐TTT in wider clinical contexts. Future replications should maintain key methodological elements, such as trained raters, standardized scoring frameworks, and uniform test materials, to ensure consistency.

Additional factors may have influenced findings. While correlation analyses examined associations between m‐TTT scores, PANSS subscale scores, chlorpromazine‐equivalent antipsychotic dosage, and educational attainment, other potential confounding variables were not assessed. Fatigue and motivation levels may have impacted task performance, and standardized assessments of these factors in future studies could help clarify their influence.

Although educational attainment was examined, prior experience in creative professions was not assessed. Occupational background may affect divergent thinking abilities, highlighting the need for future studies to collect data on participants' work history, particularly in creative fields. Additionally, all participants with schizophrenia were taking antipsychotic medications, which may have influenced their cognitive function. While no significant correlation was found between m‐TTT scores and chlorpromazine‐equivalent dosage, the long‐term effects of medication on cognitive flexibility warrant further investigation.

Despite these limitations, the findings underscore the m‐TTT's reliability and its potential for broader clinical and research applications. Refining the scoring framework, enhancing evaluator training, and standardizing guidelines may further improve scoring consistency, particularly in creative assessments. Future research should prioritize validation across diverse populations and settings to establish the m‐TTT as a versatile and robust neuropsychological assessment tool.

## CONCLUSION

The results confirmed the high interrater reliability of the m‐TTT, supporting its utility as a reliable tool for assessing divergent thinking and executive functioning. High interrater reliability was observed in both the schizophrenia and control groups, with ICC values indicating strong agreement across all scoring components. The schizophrenia group exhibited particularly high reliability, while slightly lower consistency was noted in the control group, especially in the evaluation of the creation process. These findings support the reliability and applicability of the m‐TTT in both clinical and research contexts, highlighting its potential as a valuable neuropsychological assessment tool.

## AUTHOR CONTRIBUTIONS

Yasuhisa Nakamura conceptualized and designed the study, conducted the analysis, drafted the manuscript, and serves as the corresponding author responsible for journal communication during submission, peer review, and publication. Reiko Miyamoto and Akihiro Koreki contributed to the writing and editing of the manuscript. Sachiko Anamizu and Masaru Mimura supervised the project.

## CONFLICT OF INTEREST STATEMENT

Masaru Mimura, an Editorial Board member of *Psychiatry and Clinical Neurosciences Reports*, was excluded from all editorial decision‐making regarding the acceptance of this article. All other authors declare no conflicts of interest.

## ETHICS APPROVAL STATEMENT

The study protocol was approved by the Clinical Research Ethics Committee of Nihon Fukushi University, Japan (approval No. 20‐033‐02). The study was conducted in accordance with the ethical standards outlined in the Declaration of Helsinki and Japanese national guidelines for clinical research.

## PATIENT CONSENT STATEMENT

All study participants provided written informed consent.

## CLINICAL TRIAL REGISTRATION

The trial was registered with the University hospital Medical Information Network Clinical Trials Registry (registration number UMIN000055307).

## Supporting information

Supporting information.Figure S1.

## Data Availability

The data that support the findings of this study are openly available in the Open Science Framework at https://doi.org/10.17605/OSF.IO/JPZFQ. The analysis code is not available as no programming scripts were used in this study.
