# Supplementary material for: Interrater reliability of the modified Tinkertoy test: A validation study in schizophrenia and control groups
Source: PCN Rep. 2025 Apr 3;4(2):e70094. doi: 10.1002/pcn5.70094 (PMC11968418; doi:10.1002/pcn5.70094)
Supplement: Supplementary file 1 — Supporting information.Figure S1. [file PCN5-4-e70094-s001.docx]

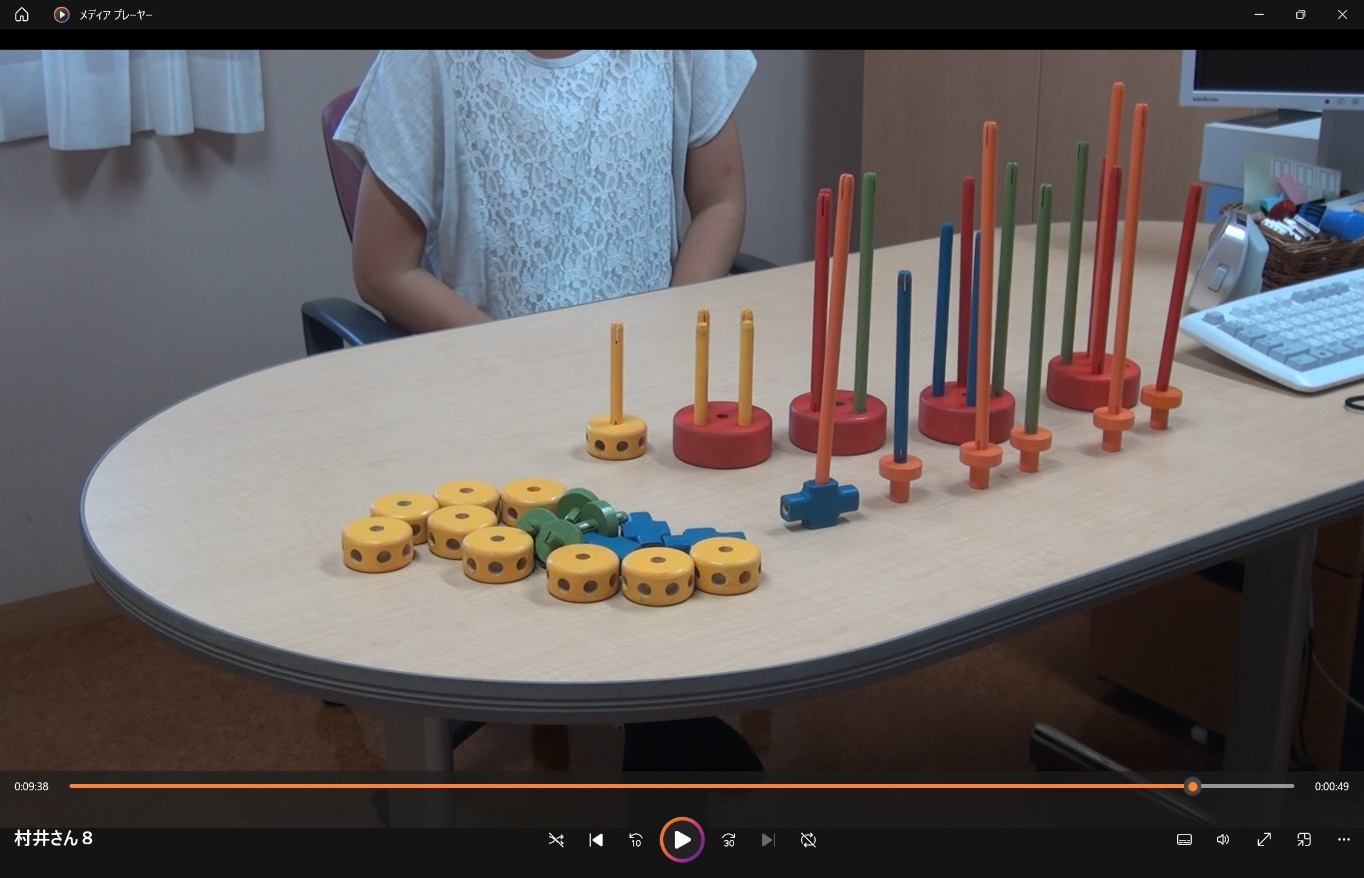

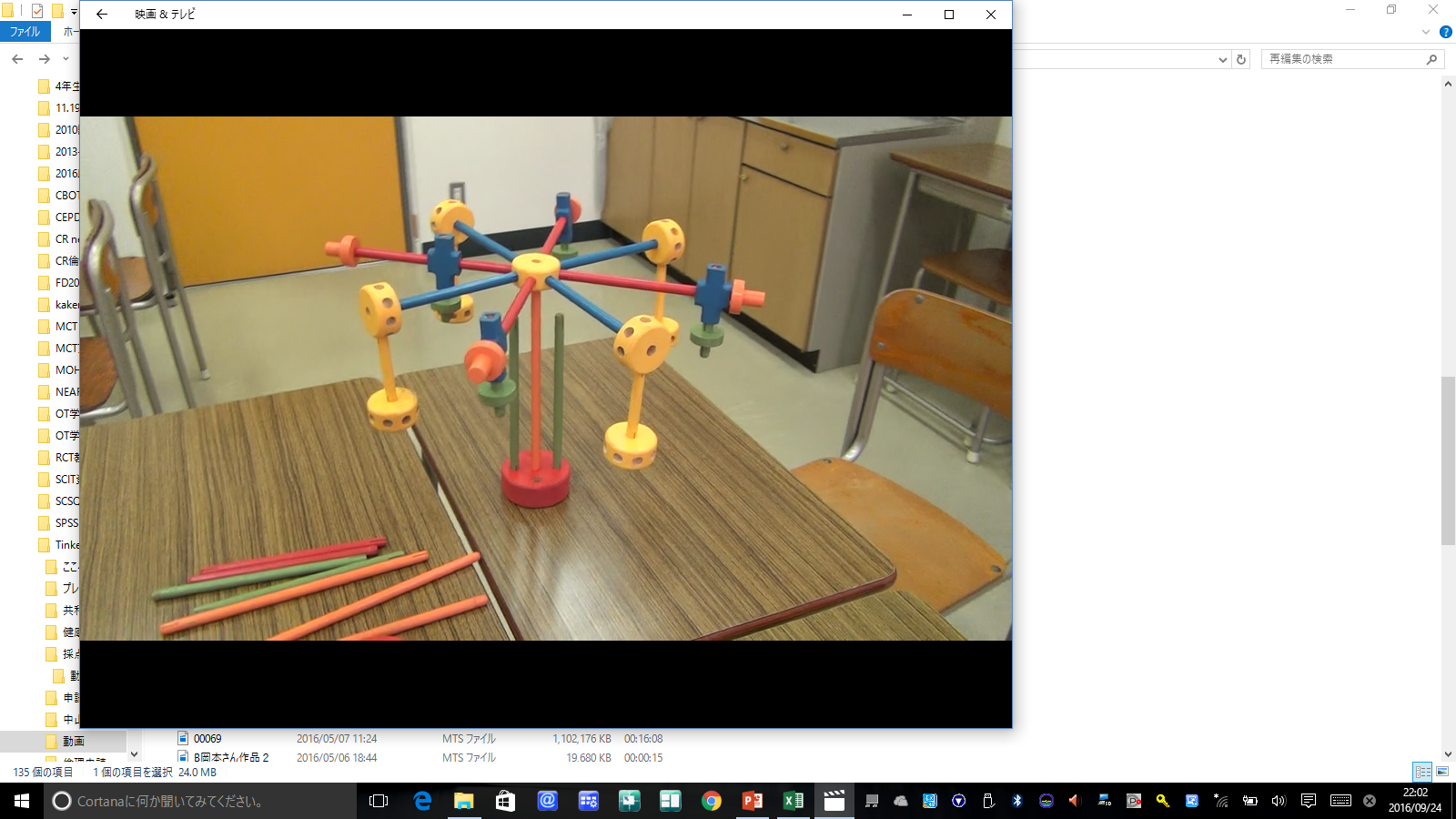


Schizophrenia group　　　　　　　　 Healthy control group

Supplementary Figure 1. Example Structures in the m-TTT Task. The left image shows a structure created by a participant in the schizophrenia group, while the right image shows a structure from a participant in the healthy control group. Differences in complexity and design approach are evident between the two.
m-TTT, Modified Tinkertoy Test
